# Influence of the Antenna Orientation on WiFi-Based Fall Detection Systems

**DOI:** 10.3390/s21155121

**Published:** 2021-07-28

**Authors:** Jorge D. Cardenas, Carlos A. Gutierrez, Ruth Aguilar-Ponce

**Affiliations:** Facultad de Ciencias, Universidad Autónoma de San Luis Potosí, Av. Chapultepec 1570, Privadas del Pedregal, San Luis Potosí C.P. 78295, Mexico; j.cardenas@ieee.org (J.D.C.); ruth.aguilar@ieee.org (R.A.-P.)

**Keywords:** fall detection, device-free, Doppler signatures, polarization, WiFi

## Abstract

The growing elderly population living independently demands remote systems for health monitoring. Falls are considered recurring fatal events and therefore have become a global health problem. Fall detection systems based on WiFi radio frequency signals still have limitations due to the difficulty of differentiating the features of a fall from other similar activities. Additionally, the antenna orientation has not been taking into account as an influencing factor of classification performance. Therefore, we present in this paper an analysis of the classification performance in relation to the antenna orientation and the effects related to polarization and radiation pattern. Furthermore, the implementation of a device-free fall detection platform to collect empirical data on falls is shown. The platform measures the Doppler spectrum of a probe signal to extract the Doppler signatures generated by human movement and whose features can be used to identify falling events. The system explores two antenna polarization: horizontal and vertical. The accuracy reached by horizontal polarization is 92% with a false negative rate of 8%. Vertical polarization achieved 50% accuracy and false negatives rate.

## 1. Introduction

Improvement on quality of life has resulted in an increased life expectancy. In 2019, the population over 65 years had a 3% growth compared to 1990. Projected population expansion indicates that elderly people will represent 16% of the world population by 2050 [1]. Much of this sector of society tend to live independently. For this reason, it is necessary to provide systems for remote healthcare and monitoring. Several systems have been developed for this purpose using well-established sensing techniques, such as acoustic sensors, video cameras, vibration sensors, and wearable devices [2,3,4,5,6]. However, an innovative technique has emerged in the last years: device-free monitoring based on radio-frequency (RF) signals. An RF signal shows fluctuations at the receiver due to absorption and reflections by moving people wandering in the proximity of the transmitting and receiving antennas [7,8]. These fluctuations can be characterized to identify activities of daily living (ADL) or to detect important sporadic events, such as the presence of burglars or the occurrence of accidents. RF monitoring systems are noninvasive because users do not need to carry a sensor and they also preserve user privacy since no image is recorded.

According to the World Health Organization, 37.3 million elderly people require medical attention each year for injuries caused by falls [9]. In contrast to ADL, like walking, running, or going up and downstairs, falls are rare or transient events. This implies that the detection of these events should be approached from a different perspective, seeking to provide prompt medical attention when a fall occurs.

In device-free monitoring, the presence of WiFi access points (APs) in indoor environments has been used to collect and classify RF signals [10,11]. Despite recent advances in the design of WiFi-based fall detection systems, most of the work has focused on developing algorithms for feature extraction and classification. Previous research has not addressed the impact of some important physical layer factors on the system’s sensing capabilities, such as the variations of the antenna response with spatial orientation. These variations stem from the angular dependence of the antenna’s radiation pattern, and also from the polarization mismatch between the transmitter ($T_{X}$) and the receiver ($R_{X}$) due to channel depolarization effects [12]. To the best of the authors knowledge, a detailed investigation of the impact of the radiation pattern’s anisotropy and antenna polarization mismatch on the performance of WiFi-based fall detection systems has not been conducted so far. However, in [13], Wang et al. analyzed the influence of antenna height on the system’s performance, and they concluded that the detection rate improves by raising the antenna height. In [14], a fall detection system was presented taking into consideration the polarization of the received signal. Nonetheless, the system was developed using Doppler radars, and the results presented there cannot be extrapolated directly to WiFi-based systems. A preliminary analysis of the effects of antenna orientation in the performance of fall detection system built around the Doppler signatures of WiFi signals was presented in [15]. There, it was empirically demonstrated that fall detection platforms are highly sensitive to antenna orientation. However, the analysis presented in [15] needs to be revisited by considering a complete implementation of feature extraction and classification algorithms to determine the accuracy of the system.

This paper presents a systematic analysis of the effects of the antenna orientation in a fall detection system based on the Doppler signatures of WiFi signals. The results of this work are intended to help researchers to determine the best antenna orientation, and to promote the design multi-polarized antennas that improve the system’s sensing capabilities without increasing the number of sensors (antennas) or the classification algorithms’ complexity. To assess these effects, an experimental fall detection platform is presented that replicates the operating conditions of a system based on the transmission of WiFi-signals. This experimental platform allows us to compute the Doppler signatures of the transmitted signal so that the features of the signal’s variations in presence of human movements can be identified and classified. A series of experiments were conducted to gather empirical data about the Doppler signatures produced by falls from a three-step ladder. The experiment is comprised of two different antenna orientation configurations: horizontal-to-horizontal (HH) and vertical-to-vertical (VV). The detection accuracy obtained for the HH configuration amounts to a 92% with a false negative rate of 8%. The VV configuration produced a 50% accuracy and a 50% false negatives rate. The obtained results provide evidence of the importance of considering the effects of antenna orientation for the design of WiFi-based fall detection systems.

The remainder of the paper has the following organization. Section 2 provides a review of related work. Section 3 details the mains components of a WiFi-based fall detection system. The mathematical model of the probe signal is presented in Section 4. In Section 5 the implementation of our experimental platform is described. Section 6 addresses the feature extraction and event classification algorithms. The results obtained are presented in Section 7. In Section 8 the results are discussed. Finally, the conclusions are given in Section 9.

## 2. Related Work

There are different sensing techniques that can be used to design and develop fall detection systems. Table 1 shows an overview of some of the most common techniques and their characteristics. Vision-based systems comprise principally digital image processing [4]. The optimization of this processing can be affected by the resolution of the images that are collected by the video recording equipment. This implies that the use of high resolution equipment is necessary, which can be expensive. Furthermore, the feature extraction process is essential for vision-based fall detection systems. First, a simple model of a human body needs to be obtained, encompassing only head, torso and legs. However, partial occlusion of a user affects the construction of such model. Additionally, camera projection artifacts must be considered to avoid a misdetection. Then a description of movement of this human body model is used for analysis. Feature analysis can be static or dynamic. In the static analysis, a morphological model of the behavior of the fall is made [16]. In the case of dynamic analysis, the movement change determines the presence of abnormal events in the general movements. This analysis allows one to identify the speed and direction of movement of the human body. In this way, the trajectories can be used to detect the behavior of a fall. Occlusions in the target’s vision also make this process difficult, making necessary to implement complex classification algorithms. Therefore, vision-based systems present drawbacks such as highly complex analysis algorithms, which require high performance computer system and a line of sight (LOS) to user compromising their privacy.

Wearable devices use accelerometers, gyroscopes, magnetometers and tilt sensors embedded in general-purpose devices that can be carried by people. These sensors operate at different frequencies, which can vary the sampling rate of the system. For this reason, it is necessary to determine the rate at which the falling event can be registered and select the sensor that best suits these conditions. However, the placement of the sensor on the human body directly affects the detection performance of the system [5,17,18]. Additionally, wearable sensors can cause discomfort for users who are not accustomed to wearing such devices.

On the other hand, environment-based systems use sensors embedded in the indoor surroundings. These sensors can be acoustic, where the transmission of sound waves is affected by changes in trajectory caused by obstacles in the environment [2]. In this way, changes in the trajectory are recorded and characterized to determine if a fall occurred. However, due to the nature of sound waves, the transmission can be degraded by the multi-path effect. Therefore, monitoring more than one indoor environment requires the implementation of a large number of sensors. The measurement of floor vibration signals through sensors such as accelerometers or piezoelectrics is another alternative for environment-based systems. Sensors mounted on the floor structure collect the variations in vibration induced by the movement of the human body to classify them. However, environment-based systems tend to be expensive due to their exhaustive implementation and the large number of sensors employed.

An alternative to all these approaches are systems based on radio frequency signals. Some proposals for the design of fall detection systems are based on Doppler radars to measure and analyze changes in signals caused by human movement [14]. However, the cost and complexity of such specialized equipment make the deployment of these monitoring systems an expensive solution. Other proposals capitalize on the ubiquity of wireless local area networks [11,21,22]. The underlying idea of these fall detection systems is to employ the signals transmitted by commercial WiFi APs as probe signals to scan the environment and detect anomalies in the received power that could be produced by a fall event. The attributes of the received signal that can be considered for anomaly detection are the received signal strength indicator (RSSI) [8], the channel state information n, and the Doppler spectral signatures [23].

A RF monitoring system based on the RSSI of WiFi signals was presented in [8]. This system analyzes the received signal to identify irregularities in the RSSI that could be produced by human movement. A limitation of this approach is that the system’s performance is sensible to changes in the environment’s characteristics. A fall detection system that considers the CSI of WiFi signals, and which is referred to as WiFall, was presented in [13]. In its simplest form (WiFall-one link), the system is composed of a single transmitting antenna and a single receiving antenna. The results presented in [13] show that WiFall-one link is capable of detecting falls, but with a rather high false alarm rate of 16%. This problem is caused by the limited angle of observation of the single antenna configuration [24]. To reduce the false alarm rate, the authors proposed an enhanced version (WiFall-two links) that incorporates a second receiving antenna as a means to reduce ambiguities due to the angle of observation. A similar system, referred to as Fall Sense, was presented in [10]. The CSI is also considered in that paper as the detection parameter, but a third receiving antenna was incorporated to increase detection accuracy and further reduce the percentage of false alarms. Nevertheless, increasing the number of antennas adds complexity to the overall detection system. A proof-of-concept of a device-free fall detection system based on the Doppler signatures of WiFi signals was presented in [23]. The results obtained in that paper suggest that such spectral signatures allow differentiation between activities and falling events.

## 3. General Structure of a WiFi-Based Fall Detection System

**Figure 1:**
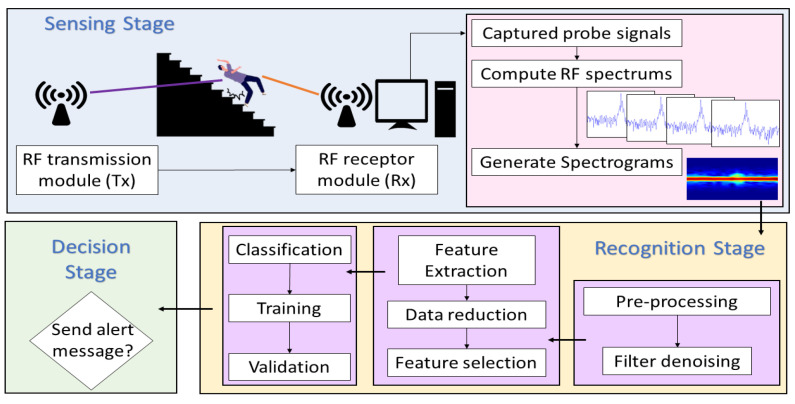
General diagram of a fall detection system based on radio frequency signals.

Fall detection by RF monitoring comprises three phases: sensing, recognition, and decision. Figure 1 shows a block diagram of these three stages for a system based on the Doppler signatures of WiFi signals. In the sensing phase, a probe signal is radiated by the transmitting antenna. This signal is not intended to carry data, it is simply a means to interact—by the mechanism of electromagnetic wave reflection—with the objects in the vicinity of the receiving antenna. If the probe signal impinges on a moving body, e.g., a falling person, then the reflected signal will suffer an apparent frequency shift due to the Doppler effect [23]. This frequency shift depends on the speed and acceleration of the moving/falling body and it therefore produces a spectral (Doppler) signature whose features can be used to detect a fall event. The Doppler signature of the received probe signal can be computed, e.g., by following the spectrogram concept [25]. Spectrograms provide information about the spectral density of a signal within a short observation time interval. In fall detection, the signals are time-varying and non-stationary. Therefore, spectrograms are excellent tools to analyze such signals.

**Figure 2:**
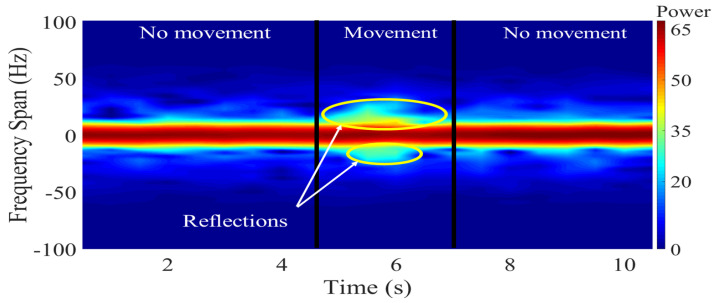
Spectrogram during a falling event.

Figure 2 shows an example of an empirical spectrogram of a constant-wave (CW) probe signal during a fall event. This figure shows static scenarios within the first 4.5 s and the last 3 s where no human movement is observed. The power density of the received signal is therefore concentrated in the origin. On the other hand, a fall occurs during a time interval spanning 4.5 to 7 s. The acceleration of the falling body produces a frequency drift of received power. Such a frequency drift can be analyzed to identify characteristic patterns of fall events. The example shown in Figure 2 considers motion of a single body. This scenario is relevant for fall detection of elderly people living independently, as these individuals have no one around and therefore rely on an automatic wireless monitoring system to get assistance in case of a fall. The single-person scenario shown in Figure 2 may not be relevant for ADL applications, in which several persons, each producing a different Doppler signature, could be around the receiving antenna. For these latter applications, the goal of the system is to isolate and identify the activities realized by each person.

Classification stage is performed after acquiring a spectrogram. In this stage, the signals are classified based on the information extracted from their features. The feature extraction is based on finding the dataset that allows quantitatively differentiating if the signals correspond to a falling event or other activities. A dimensionality-reduction technique widely used for feature extraction is Principal Component Analysis (PCA). Spectrograms are high-dimensional data that can be further explored into a lower dimensional space that retains most of the information. Therefore, PCA is applied to the acquired spectrogram. The extracted principal components can be used by classification algorithms as features to recognize falling events. However, the number of techniques and approaches that can be used for data analysis at this stage is very extensive. The feature selection in RF-WiFi signals and the techniques used for the training phases must be balanced to the needs of the detection system. In [21], Di Domenico et al. explore different training approaches for motion sensing platforms based on RF signals and Doppler spectrum. Furthermore, they show that it is necessary to establish the necessary trade-offs in terms of performance, time-consuming, complexity, and the number of features in these applications. A fall detection platform implemented in [26] shows the performance of different classification algorithms using six features extracted of the instantaneous Doppler frequency of a RF signal. Detection accuracy was increased by implementing different classification algorithms such as artificial neural network (ANN), K-nearest neighbors (KNN), quadratic support vector machine (QSVM), or ensemble bagged tree. Fall detection systems have been proposed that implement novel classification algorithms, e.g., adversarial data argumentation [27], recurrent neural networks [28], among others, which positively impact accuracy rates. However, this area has been under intensive research, while the sensing has been vaguely explored. This is in spite of the fact that the quality of the signal features are directly related to the sensing capabilities of the platform.

Finally, the decision stage comprises decision algorithms intended to issue alerts when falls occur. This stage processes the data in real-time to determine when a fall is detected. In this way, the user will receive medical attention as soon as possible. The emergency alert is part of the final system process and therefore represents a device-level implementation. Therefore, the decision stage is outside the scope of this work and remains as future work.

## 4. Signal Model

**Figure 3:**
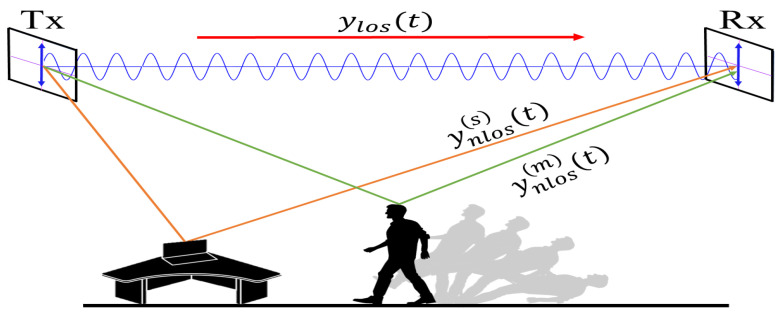
Schematic propagation of an RF signal in a real test scenario.

The antenna orientation influence on sensing and feature extraction stages of a WiFi-based fall detection system is analyzed in this paper by considering the transmission of a CW probe signal in an indoor environment. This signal could be one of the pilot signals defined within the WiFi physical protocol data unit [29], or a specific purpose signal transmitted by the APs when there is not an active communication session with a user node. We assume the probe signal is received by another WiFi device that acts as the system’s sensor and which is located in a different position than the AP. Furthermore, we assume that the received probe signal comprises a line-of-sight (LOS) component and a non-LOS (NLOS) component due to single reflections from *K* static objects (e.g., walls and furniture) and a moving object (falling person), as illustrated by Figure 3.

The received signal can be written in the complex base-band equivalent as:

(1) $$\begin{array}{ccc} y(t) & = & n_{\sigma }\left(t\right)+y_{\mathrm{los}}+y_{\mathrm{nlos}}^{\left(s\right)}+y_{\mathrm{nlos}}^{\left(m\right)}\left(t\right). \end{array}$$

In this equation, $n_{\sigma }\left(t\right)\in C$ is additive white Gaussian noise with power density $\sigma^{2}$; C stands for the set of complex-valued numbers. The LOS component of y(t) is a time-invariant signal modeled by

(2) $$\begin{array}{cc} y_{\mathrm{los}} & =E\left({\vec{u}}_{0}\right)V\left(-{\vec{u}}_{0}\right)X_{\mathrm{los}}g_{0}e^{-j\theta_{0}} \end{array}$$

where $E\left({\vec{u}}_{0}\right)\in R_{0}^{+}$ is the transmitting antenna’s radiation pattern evaluated in the direction of the unit vector, ${\vec{u}}_{0}$, that points toward the $R_{X}$ from the position of the $T_{X}$; the symbol $R_{0}^{+}$ stands for the set of non-negative real numbers. In turn, $V\left(-{\vec{u}}_{0}\right)\in R_{0}^{+}$ is the vector effective length of the receiving antenna evaluated in the direction from the $R_{X}$ to the $T_{X}$. Signal attenuation due to path loss is modeled by $g_{0}\in R_{0}^{+}$, and $\theta_{0}=2\pi D_{0}/\lambda$ is a phase shift that depends on the signal’s wavelength λ and the distance $D_{0}$ between the $T_{X}$ and the $R_{X}$. The absolute value of $X_{\mathrm{los}}\in C$ is the polarization loss factor (PLF), which characterizes the polarization mismatch between the transmitting and receiving antennas. We note that $|X_{\mathrm{los}}|\leq 1$, and $|X_{\mathrm{los}}|=1$ if the antennas are co-polarized, whereas $|X_{\mathrm{los}}|=0$ if they are cross-polarized.

The NLOS component of y(t) due to single reflections from the *K* static objects is characterized as a time-invariant signal

(3) $$\begin{array}{cc} y_{\mathrm{nlos}}^{\left(s\right)} & =\sum_{k=1}^{K}E\left({\vec{u}}_{k}^{\left(s\right)}\right)V\left({\vec{v}}_{k}^{\left(s\right)}\right)X_{\mathrm{nlos}}^{\left(s,k\right)}g_{k}^{\left(s\right)}e^{-j\left[\phi_{k}^{\left(s\right)}+\theta_{k}^{\left(s\right)}\right]} \end{array}$$

where ${\vec{u}}_{k}^{\left(s\right)}$ and ${\vec{v}}_{k}^{\left(s\right)}$ are unit vectors that point toward the *k*th static reflector from the position of the $T_{X}$ and the $R_{X}$, respectively. The scalar variable $g_{k}^{\left(s\right)}\in R_{0}^{+}$ accounts for the combined effects of path loss and signal attenuation due to the interaction with the $k_{th}$ reflector. Likewise, $\phi_{k}^{\left(s\right)}$ accounts for the phase shift that results from such an interaction. The phasor $g_{k}^{\left(s\right)}\exp\left\{j\phi_{k}^{\left(s\right)}\right\}$ can therefore be described as a weighted version (weighted by the path loss) of the reflection coefficient that characterizes the reflection of electromagnetic waves from lossy dielectrics [30]. The phase $\theta_{k}^{\left(s\right)}$ is given as $\theta_{k}^{\left(s\right)}=2\pi D_{k}^{\left(s\right)}/\lambda$, where $D_{k}^{\left(s\right)}$ is the total length of the path among the $T_{X}$, the $k_{th}$ reflector, and the $R_{X}$. The absolute value of $X_{\mathrm{nlos}}^{\left(s,k\right)}\in C$ also characterizes the PLF between $T_{X}$ and $R_{X}$. However, the difference between $X_{\mathrm{nlos}}^{\left(s,k\right)}$ and $X_{\mathrm{los}}$ is that the former incorporates effects of depolarization caused by the reflection from the $k_{th}$ static object [12]. In turn, the NLOS component of y(t) stemming from the reflection off the falling person is modeled by

(4) $$\begin{array}{cc} y_{\mathrm{nlos}}^{\left(m\right)}\left(t\right) & =E\left({\vec{u}}^{\left(m\right)}\right)V\left({\vec{v}}^{\left(m\right)}\right)X_{\mathrm{nlos}}^{\left(m\right)}g^{\left(m\right)}e^{-j\left[\phi^{\left(m\right)}+\theta^{\left(m\right)}-\varphi^{\left(m\right)}\left(t\right)\right]}. \end{array}$$

Except for the time-varying phase $\varphi^{\left(m\right)}\left(t\right)$, the variables on the right-hand side of (4) are defined as their counterparts with the (s) superscript but considering the moving (falling) person as the reflector. The phase $\varphi^{\left(m\right)}\left(t\right)$ is given as $\varphi^{\left(m\right)}\left(t\right)=2\pi \int_{0}^{t}\nu_{d}\left(x\right)\;dx$, where $\nu_{d}\left(t\right)$ is the total time-varying Doppler frequency shift caused by the velocity of the person during a fall [31]. Assuming that the person falls with a constant acceleration, $\varphi^{\left(m\right)}\left(t\right)$ can be characterized as a quadratic phase $\varphi^{\left(m\right)}\left(t\right)=2\pi \left[\nu_{s}t+{\hat{\nu }}_{a}t^{2}/2\right]$, where $\nu_{s}$ is the Doppler frequency shift produced by the initial speed of the falling person, and ${\hat{\nu }}_{a}$ is a frequency drift having units of hertz per second. Thereby, the total Doppler shift can be written as

(5) $$\begin{array}{c} \nu_{d}\left(t\right)=\nu_{s}+\nu_{a}\left(t\right) \end{array}$$

with $\nu_{a}\left(t\right)={\hat{\nu }}_{a}t$ accounting for the component of the time-varying Doppler shift due to acceleration [32].

The spectrogram of the received probe signal is given as

(6) $$\begin{array}{cc} S(t^{\prime };\nu ) & =|Y\left(t^{\prime };\nu \right){|}^{2} \end{array}$$

where $t^{\prime }$ is an arbitrary observation time, $Y\left(t^{\prime };\nu \right)=\int_{-\infty }^{\infty }y\left(t\right)w\left(t-t^{\prime }\right)e^{-j2\pi \nu t}\;dt$ is the short-time Fourier transform (STFT) of y(t), and w(t) is an even and positive windowing function of unit energy [25]. For the signal defined by (1)–(4), we have

(7) $$\begin{array}{c} Y\left(t^{\prime };\nu \right)=N_{\sigma }\left(t^{\prime };\nu \right)+A^{\left(s\right)}W\left(t^{\prime };\nu \right)+Y_{\mathrm{nlos}}^{\left(m\right)}\left(t^{\prime };\nu \right) \end{array}$$

where $W(t^{\prime };\nu )$, $Y_{\mathrm{nlos}}^{\left(m\right)}\left(t^{\prime };\nu \right)$, and $N_{\sigma }\left(t^{\prime };\nu \right)$ are the STFT of w(t), $y_{\mathrm{nlos}}^{\left(m\right)}\left(t\right)$, and the noise signal, respectively, whereas $A^{\left(s\right)}=y_{\mathrm{los}}+y_{\mathrm{nlos}}^{\left(s\right)}$. In this paper, we consider a Gaussian window function $w\left(t\right)={\left(\sqrt{\pi }\sigma_{s}\right)}^{-1/2}e^{-t^{2}/\left(2\sigma_{s}^{2}\right)}$, with spread parameter $\sigma_{s}$ [32], due to its good frequency resolution and reduced spectral leakage. This window results in wide peaks and low sidelobes, which allows a better frequency resolution. The Gaussian window is widely used in applications of time-frequency analysis. For a Gaussian pulse window, it can be shown (see [32]) that

(8) $$\begin{array}{cc} W(t^{\prime };\nu ) & =\sqrt{2\sigma_{s}\sqrt{\pi }}e^{2\pi [j\nu t^{\prime }-\pi {\left(\sigma_{s}\nu \right)}^{2}]} \end{array}$$

(9) $$\begin{array}{cc} Y_{\mathrm{nlos}}^{\left(m\right)}\left(t^{\prime };\nu \right)\; & =\frac{A^{\left(m\right)}}{\pi^{\frac{3}{4}}\sigma_{y}\sqrt{2\sigma_{s}}}e^{-\frac{{\left[\nu -\nu_{d}\left(t^{\prime }\right)\right]}^{2}}{2\sigma_{y}^{2}}}e^{j[\varphi^{\left(m\right)}\left(t^{\prime }\right)-\varphi^{\left(m\right)}-\theta^{\left(m\right)}-2\pi \nu t^{\prime }]} \end{array}$$

where $\sigma_{y}^{2}=\left[1-j2\pi \sigma_{s}^{2}{\hat{\nu }}_{a}\right]/{\left(2\pi \sigma_{s}\right)}^{2}$, and

(10) $$\begin{array}{cc} A^{\left(m\right)} & =E\left({\vec{u}}^{\left(m\right)}\right)V\left({\vec{v}}^{\left(m\right)}\right)X_{\mathrm{nlos}}^{\left(m\right)}g^{\left(m\right)}. \end{array}$$

By substituting (7) into (6), we find

(11) $$\begin{array}{cc} S(t^{\prime };\nu ) & =S_{n}\left(t^{\prime };\nu \right)+{\left|A^{\left(s\right)}\right|}^{2}S_{w}\left(t^{\prime };\nu \right)+S_{y}^{\left(m\right)}\left(t^{\prime };\nu \right)+C\left(t^{\prime };\nu \right). \end{array}$$

In this equation,

(12) $$\begin{array}{cc} S_{n}\left(t^{\prime };\nu \right) & =|N_{\sigma }\left(t^{\prime };\nu \right){|}^{2} \end{array}$$

(13) $$\begin{array}{cc} S_{w}\left(t^{\prime };\nu \right) & =|W\left(t^{\prime };\nu \right){|}^{2}=2\sigma_{s}\sqrt{\pi }e^{-{\left[2\pi \sigma_{s}\nu \right]}^{2}} \end{array}$$

(14) $$\begin{array}{cc} S_{y}^{\left(m\right)}\left(t^{\prime };\nu \right) & ={\left|Y_{\mathrm{nlos}}^{\left(m\right)}\left(t^{\prime };\nu \right)\right|}^{2}=\frac{{\left|A^{\left(m\right)}\right|}^{2}}{\sqrt{2\pi }\gamma }e^{-\frac{{\left[\nu -\nu_{d}\left(t^{\prime }\right)\right]}^{2}}{2\gamma^{2}}} \end{array}$$

for $\gamma^{2}=\left[1+{\left(2\pi \sigma_{s}^{2}{\hat{\nu }}_{a}\right)}^{2}\right]/\left[2{\left(2\pi \sigma_{s}\right)}^{2}\right]$, and $C(t^{\prime };\upsilon )$ is a complex-valued function accounting for the cross-products among $N_{\sigma }\left(t^{\prime };\nu \right)$, $A^{\left(s\right)}W\left(t^{\prime };\nu \right)$, $Y_{\mathrm{nlos}}^{\left(m\right)}\left(t^{\prime };\nu \right)$ and their corresponding complex conjugates [32].

Equation (11) shows that the spectrogram of the received signal is composed of two Gaussian pulses plus a noise term and artifacts resulting from the cross-products of the $C(t^{\prime };\upsilon )$ function. The Gaussian pulse $|A^{\left(s\right)}{|}^{2}S_{w}\left(t^{\prime };\nu \right)$ is an origin-centered pulse whose amplitude is scaled by the combination of $y_{\mathrm{los}}\left(t\right)$ and $y_{\mathrm{nlos}}^{\left(s\right)}\left(t\right)$. The second Gaussian pulse, $S_{y}^{\left(m\right)}\left(t^{\prime };\nu \right)$ is centered at the Doppler frequency $\nu_{d}\left(t\right)$ and it amplitude depends on the antenna characteristics involved in the definition of $A^{\left(m\right)}$ (see (10)).

**Figure 4:**
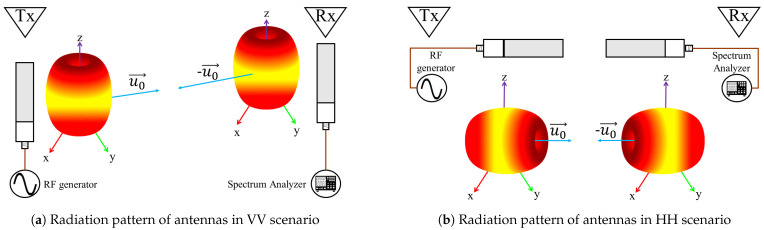
Radiation pattern and polarization of antennas configuration in both scenarios.

The combined spectrogram of the LOS and static-reflectors NLOS signals does not contribute meaningful information for fall detection. This is because such signals remain unchanged during a fall event as they are not influenced by the Doppler effect. All the relevant information about the direction and speed of the fall is embedded in the spectrogram of the moving-reflector NLOS signal through the dependence of $S_{y}^{\left(m\right)}\left(t^{\prime };\nu \right)$ on the time-varying Doppler shift $\nu_{a}\left(t\right)$. However, a proper detection of $S_{y}^{\left(m\right)}\left(t^{\prime };\nu \right)$ depends not only on a good match between the polarization of both antennas, but also on a good alignment between the radiation pattern of the transmitting antenna and the vector effective length of the receiving antenna. The alignment of the radiation pattern and polarization during transmission depends on the antenna orientation, as illustrated in Figure 4. By changing the position of $T_{X}$ and $R_{X}$, mismatches occur and change the effective radiation zones where the signal arrives [33,34]. Misalignments that decrease $S_{y}^{\left(m\right)}\left(t^{\prime };\nu \right)$ strength can be mitigated by varying the orientation of the antennas and analyzing changes in the spectrogram of the received signal.

From an analytical perspective, it is a complex process to analytically compute the optimal antenna placement and orientation that maximize the value of ${\left|A^{\left(m\right)}\right|}^{2}$ for any given indoor environment. However, to gain insight into the influence of the directional characteristics of the antenna system, we have implemented a WiFi-like experimental platform for fall detection that allows us to empirically analyze the effects of the antenna misalignment in the system’s performance. Using the implemented experimental platform, we conducted a series of experiments in two scenarios: VV (Figure 4a) and HH (Figure 4b). Through the use of classification algorithms, we analyze and determine the orientation of the antennas that provide spectrograms with a higher resolution of the fall data. The implementation of our experimental platform is described in the following section.

## 5. System Design

### 5.1. Hardware Setup

The $T_{X}$ module of our experimental platform comprises a RF signal generator connected to a monopole antenna whose radiation pattern is omnidirectional in any plane perpendicular to the monopole’s axis. The $R_{X}$ module is composed of a monopole antenna connected to a spectrum analyzer. Both antennas were empirically characterized to ensure a proper coupling with the measurement equipment. The results are shown in Table 2. The antennas obtained the best parameters at a frequency of 2.42 GHz using an RG-58 coaxial cable. This operating frequency is within the range of commercial WiFi systems (IEEE 802.11b). Our $T_{X}$ module emits a pure tone with this frequency. In our case, we used a Keysight N9310A RF generator with a transmission power of 20 dBm. The transmitted probe signal was received by a second monopole antenna with the same characteristics. Afterward, the signal was processed by a spectrum analyzer. We used a spectrum analyzer FieldFox N9912A. This analyzer allowed us to capture a series of snapshots of the signal. Thereby, it was possible to record the Doppler signatures of the probe signal caused by fall event. The configuration used for the capture is shown in Table 3. This configuration allowed a balanced trade-off between the signal resolution and the sweep time.

**Figure 5:**
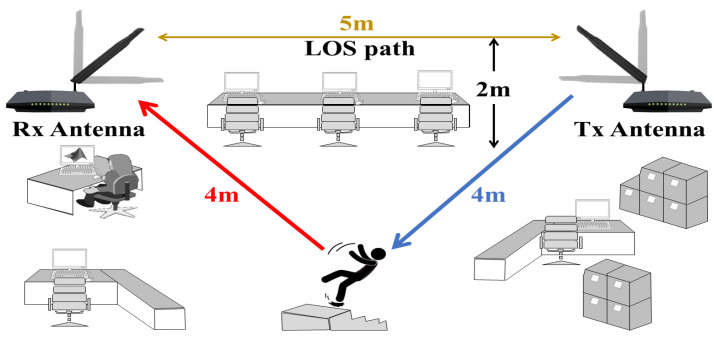
Schematic of the test scenario.

**Figure 6:**
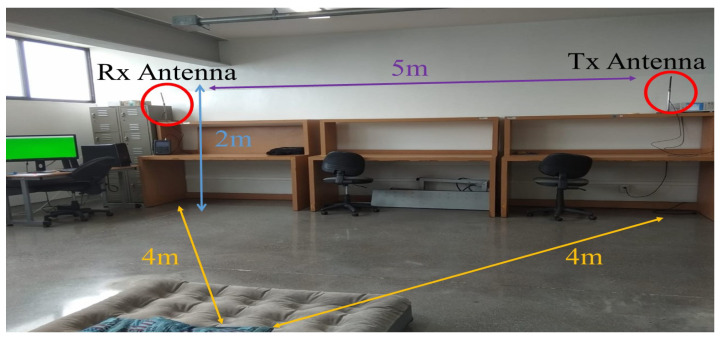
Indoor environment for the test experimentation.

The experiments with falls were conducted in an indoor environment with fixed furniture. Figure 5 shows the position of the transmitting and receiving modules during the experiments. The $T_{X}$ module was installed in the top right corner and the receiver in the top left corner of the room 5 m apart from each other. Both antennas were located at a height of 2 m and maintain an unobstructed LOS. This indoor environment corresponded to an office room and had a ladder in the center of the room where falling was performed (Figure 6). The platform allowed changing the antenna orientation according to test VV and HH settings.

### 5.2. Experimental Protocol

Our experimentation protocol sought to replicate the typical conditions of indoor environments that independent elderly people face daily. To that end, it was necessary to consider that the experiments had to be developed in the presence of a single individual in the environment and surroundings. Furthermore, the inclusion of other activities in the protocol such as running, jumping, or waving the arms is beyond the scope of the work. On the other hand, the activities carried out daily such as crouching down, raising the arms, or getting dressed did not produce an acceleration of the body compared to that of a fall. Then, our protocol focused on the static and falling state of the participants.

The empirical data were collected for a group of people falling from different heights in the VV and HH scenario. The participant’s age ranged from 23 to 48 years old. Furthermore, the group was composed of three males and three females. All participants were previously evaluated to validate that they were in good physical condition. The physical characteristics of each individual are shown in Table 4. Due to the nature of experimentation, selecting subjects with a higher age range could put their health at risk.

The experimentation protocol consisted of a sequence of falls from a height of 59 cm. This height was determined according to the specifications of the third step of a ladder. In total, the participants performed a sequence of 10 falls for each scenario.

The experiment started with a participant placed immobile on the ladder. A visual and audible signal was configured to notify the participants of the beginning and end of the experimentation. At the beginning of the test, the participants remained in a static state until a signal is sent indicating the start of the falling movement. The participants were also instructed to remain immobile once the falling movement has been performed. The notifications of preparation, start, and end of the tests were triggered automatically by the system to avoid the presence of more people near the test scenario. Finally, the signal notified the end of the test to the subjects. The information collected in the experiments was used in the subsequent stages of the system to identify falling events.

## 6. Feature Extraction and Classification Algorithm

The classification stage in our platform involved the pre-processing, extraction, and classification of the acquired signals. In our system, the analyzed features must contain the information necessary to determine the antenna orientation with which the misalignment in fall detection could be mitigated. Our signal model indicated that frequency drifts recorded in the spectrograms as Doppler signatures could be used as detection features. The considered scenarios as described earlier consisted of VV and HH antenna disposition. Each set was composed of 60 spectrograms computed during the experiments. In order to extract the most relevant information from the spectrograms, a dimensionality-reduction technique was employed.

We selected a principal component analysis (PCA) algorithm given its wide application in data analysis problems [35,36,37,38,39,40,41]. PCA is based on singular value decomposition (SVD) technique, which generates a low-dimensional approximation of a high-dimensional data set in terms of its dominant patterns [42]. The PCA algorithm reduces the dimension of the features and orders them hierarchically. Furthermore, this technique can be used to represent the correlation of high-dimensional data through a coordinate system.

The first step in the PCA algorithm is to pre-process the calculated spectrograms. The pre-processing in our system first reduced the frequency span of the spectrums to a total bandwidth of 200 Hz. This was because the frequency drifts of the probe signal did not exceed this bandwidth value. Moreover, a denoising filter was applied to smooth the additive white Gaussian noise in the CW probe signal. The data set was then organized as a matrix $X\in C^{m\times n}$, where the rows $x_{k}\in C^{n}$ contained the instantaneous spectrum of the probe signal received during the $k_{th}$ time instant, k∈{1,2,…,m}. The next step was to compute the mean of the *n* elements of row $x_{k}$ and subtract it from *X*. This standardization process resulted in the mean-subtracted data *B*:

(15) $$B=X-\bar{X},$$

where $\bar{X}$ is the matrix of means. Then, it is necessary to compute the covariance matrix of the rows of *B* given by

(16) $$C=B^{T}B.$$

The covariance matrix describes all relationships between pairs of variables in the data set [43]. In the next step, the eigen-decomposition of the covariance matrix *C* is computed and resulting:

(17) CV=VD,

in this case *V* and *D* correspond to the eigenvectors and eigenvalues respectively. Finally, the matrix of principal components is defined as,

(18) T=BV,

where *T* corresponds to the matrix of principal components. Furthermore, matrix *B* can be defined by the SVD as:

(19) $$B=U\Sigma V^{T}$$

in this case, $U\in C^{n\times n}$ and $V\in C^{m\times m}$ are unit matrices with orthonormal columns, and $\Sigma \in R^{n\times m}$ is a matrix with non-negative real integers on the diagonal and zeros off the diagonal [42]. Therefore, when substituting (19) in (18), the matrix of principal components would be given by:

(20) T=UΣ

**Figure 7:**
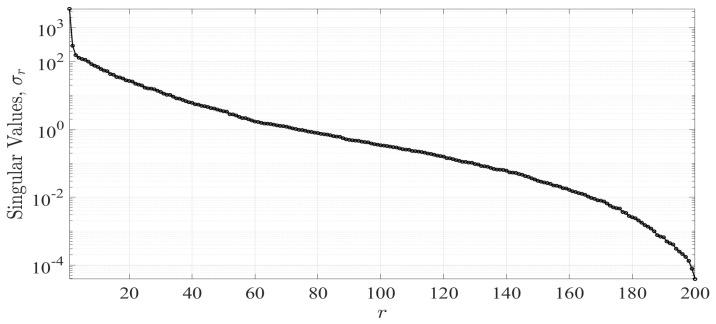
Principal components of the frequency of a Doppler spectrum.

The numerical values resulting from the PCA for the spectrogram data set could be represented in a coordinate system. These values had a logarithmic distribution as in Figure 7. In our case, the resulting number of principal components *r* corresponds to the 200 frequency values measured for each instantaneous Doppler spectrum captured during the experiments. Additionally, $\sigma_{r}$ are the elements on the diagonal of Σ that corresponded to the singular values. Furthermore, the first three singular values $\sigma_{r}$ resulting from the PCA were taken as the features of the received signal. This was because the greater variance of the values of our Doppler spectrums was concentrated in these components. The first principal component in our case generally comprised 99%, the second 0.01%, and the third 0.002%. The rest of the components could be omitted because they represented a minimum of information loss. Therefore, our system used these components as the particular features of each spectrum that were used in the classification process.

The resulting low-dimension feature space was used for classification. At this stage, it was necessary to classify the extracted features into different classes. Support Vector Machine (SVM) was employed due to its balance between complexity and accuracy. Additionally, SVM has demonstrated its effectiveness in fall detection [13,26]. SVM classification allow us to measure the impact of the antenna orientation in the accuracy of the overall fall detection system. SVM algorithms are learning machines that analyze and recognize patterns in a set of features. This supervised learning model allows us to distinguish between different classes. Then, we can determine if the data introduced belong to a falling event or a static scenario. The implementation was developed using Matlab™ classification learner toolbox. Based on the feature space generated by PCA, we selected a quadratic kernel to maximize the geometric margin of our training data.

We evaluated the performance of the classification using a re-sampling process known as the cross-validation method. The data introduced were randomly shuffled and divided into *K* folds. The first fold was used for validation and the rest for training. When the process concluded, the performance score was preserved and the model was discarded. Afterward, the process was iterated *K* times. Therefore, the model could be trained K−1 times. Finally, the performance scores were averaged to reduce randomness and obtain an accurate result. Performance scores were obtained using five folds of the cross-validation method.

## 7. Results

**Figure 8:**
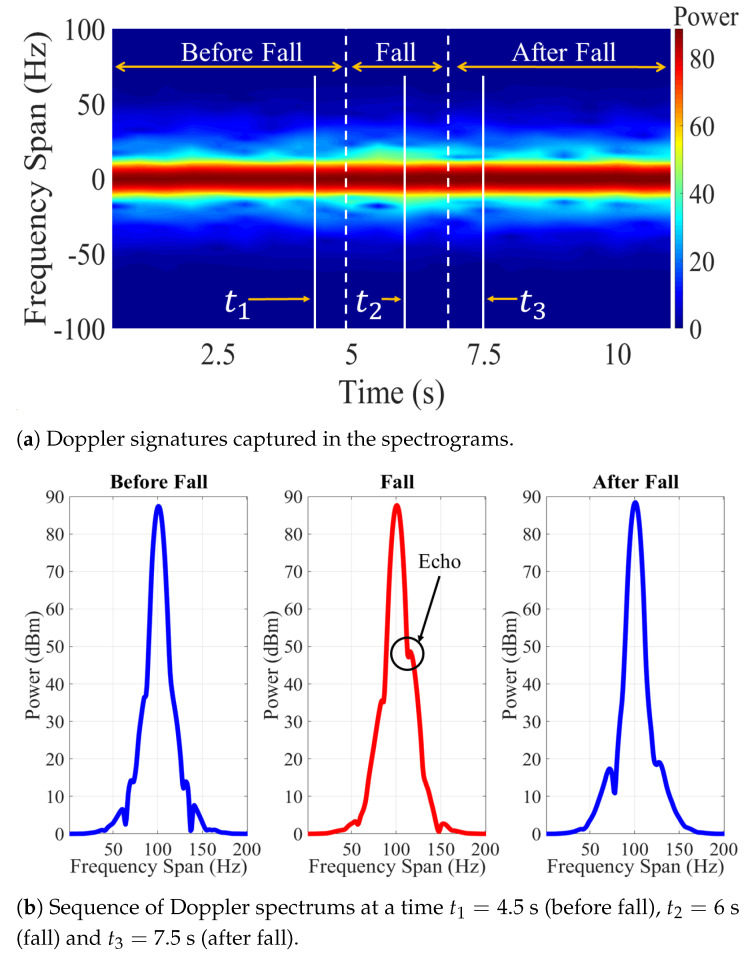
Experimental results using VV scenario setup: (**a**) spectrogram, (**b**) sequence of spectrums.

**Figure 9:**
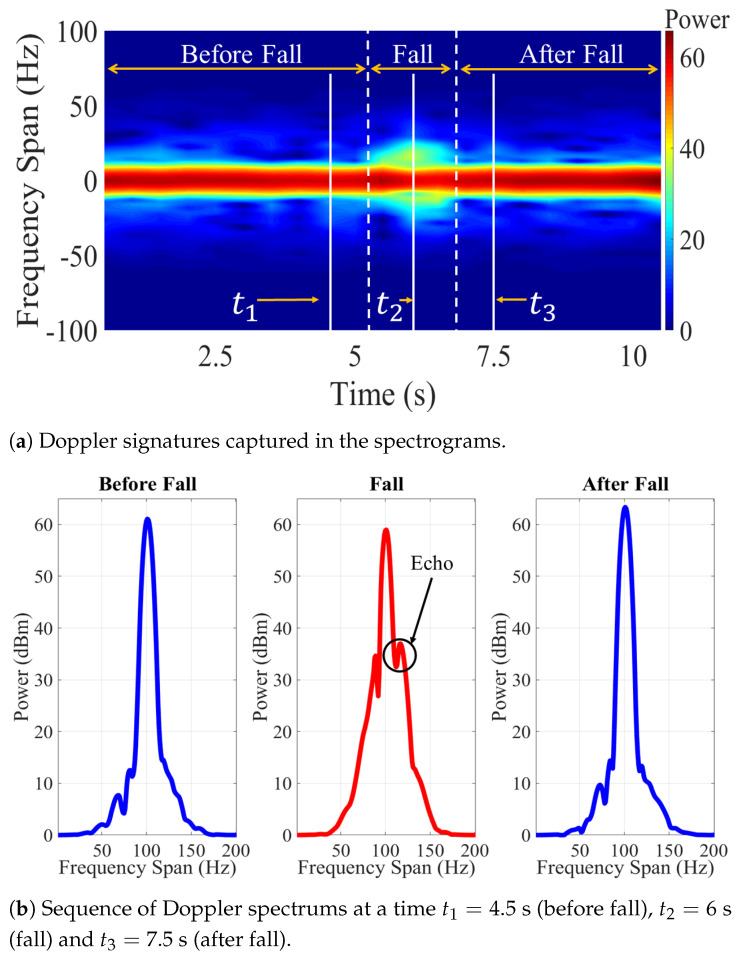
Experimental results using HH scenario setup: (**a**) spectrogram, (**b**) sequence of spectrums.

The results obtained by our platform allowed us to assess the impact of antenna polarization in the fall detection rate. The pre-processing results of the signals are shown in Figure 8 and Figure 9. Frequency dispersions caused by human movement during tests were notable in each spectrogram time sample.

Figure 8 shows the results obtained during one of the experiments with the VV orientation of the antennas. The falling event was recorded at 6 s of the experimentation. In the spectrogram of Figure 8a, the Doppler signatures captured during falling caused a dispersion of the probe signal.

On the other hand, Figure 8b shows a sequence of the frequency spectrums before, during, and after the falling event. In the spectrum corresponding to falling event, echoes could be observed in the carrier signal. These echoes corresponded to the fluctuations produced by the Doppler effect. Therefore, the configuration with the antennas in VV orientation achieved to capture the dispersions caused by the falls and the data could be used in the recognition stage.

The dataset of the HH scenario was processed following the same methodology. The results of the experiments with this orientation of the antennas are shown in Figure 9. In this case, the falling event was also recorded at 6 s. In Figure 9a the Doppler signatures at 6 s are distinguishable from the rest of the time samples recorded during the experimentation. The data showed a clear difference between the two datasets. Figure 9b shows a higher amplitude echo in the HH scenario compared to the VV scenario shown in Figure 8b.

Both datasets captured dispersions caused by falling events. It is important to mention that both experiments were carried out under the same conditions and with the same angle of observation of the antennas. Nevertheless, the Doppler signatures in the HH case were more prominent. Therefore, the most prominent dispersion was achieved with this orientation.

**Figure 10:**
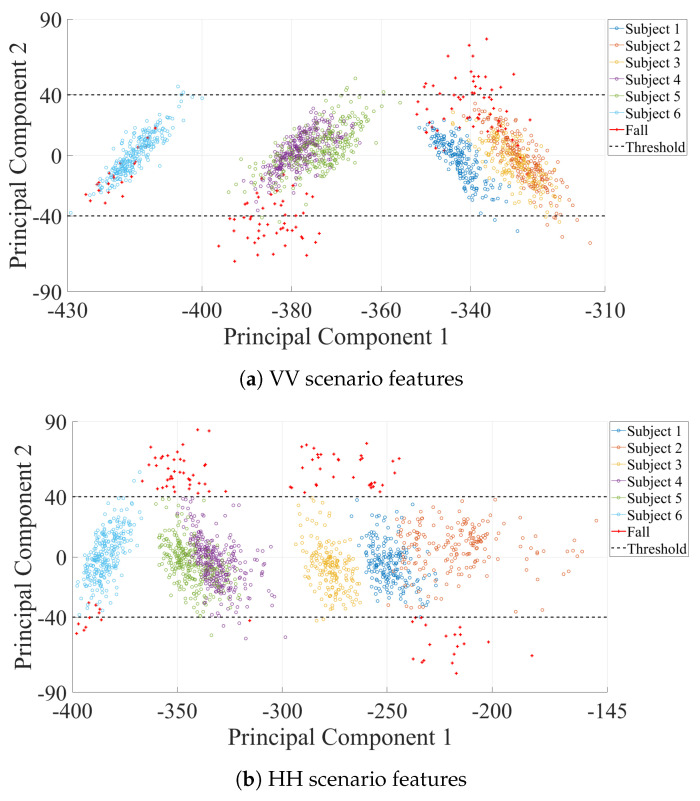
Projection of the first and second principal components.

To evaluate the impact of the antenna orientation, the frequency was taken as the feature of the Doppler spectrums. The feature space was reduced using the PCA for both scenarios. Figure 10 shows the comparison of the feature space obtained in each of the scenarios using a projection of the first two principal components. Figure 10a contains the numerical values obtained with the data from the VV scenario. The crosses in Figure 10a marks the identified falls. The rest of the features corresponded to non-movement period and are shown as dots. The distribution of the data of both classes shows in multiple cases showed similarities between their numerical values. Figure 10a shows a low separability between the non-movement and the falling events features, this made the classification process between both classes difficult.

On the other hand, the projection of the features of the HH scenario in Figure 10b shows a contrasting distribution compared to that of the VV scenario. Most of the features that corresponded to falls had numerical values that could be differentiated from the rest. This separation between both classes determined that the features extracted from the spectrum where a fall occurred using the HH orientation of the antennas obtained a greater variability than the rest.

**Figure 11:**
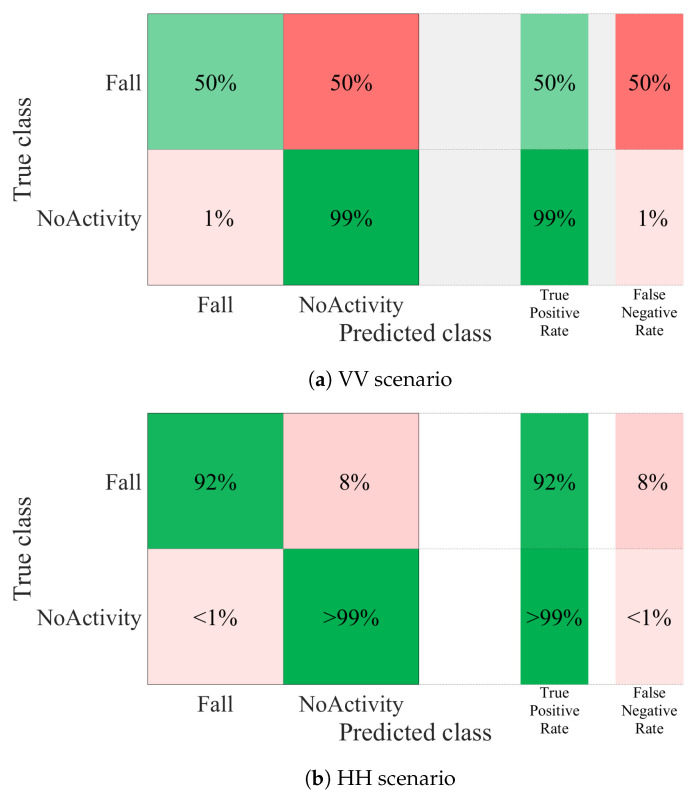
Confusion matrix of the SVM algorithm.

Classification was performed with SVM and the results are shown in Figure 11. Using the dataset of the VV scenario, an accuracy of 50% in the detection of falls was achieved (Figure 11a). Therefore, the false-negative rate was also 50%. This represents a high rate of false alarms in the system. The low percentage of accuracy was attributed to the sensing capability of the system when using a VV orientation of the antennas. After analyzing the principal components of the received signal in this scenario, it was shown that they had low variability and negatively impact the classification algorithm.

The results for the HH scenario dataset are shown in Figure 11b. In this case, the accuracy percentage increased. The evaluation results showed a 92% accuracy in the detection of falls. The false negative rate decreased to 8%. This indicated that the sensing capabilities of the system could be increased by analyzing the effects related to the orientation of the antennas. This may represent a solution to the limitation of systems based on RF-WiFi signals without the need to increase the number of sensors in the indoor environment.

**Figure 12:**
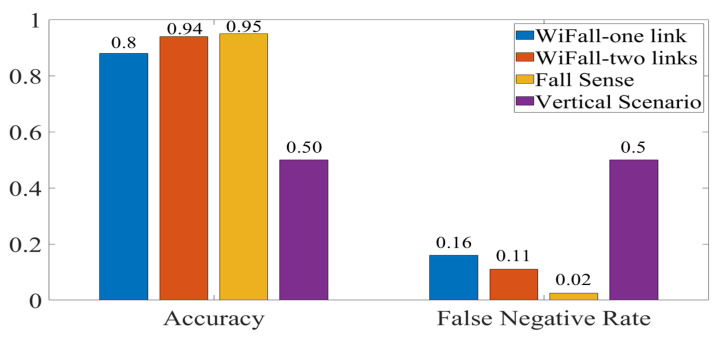
Comparison of Wifall, FallSense and VV scenario.

**Figure 13:**
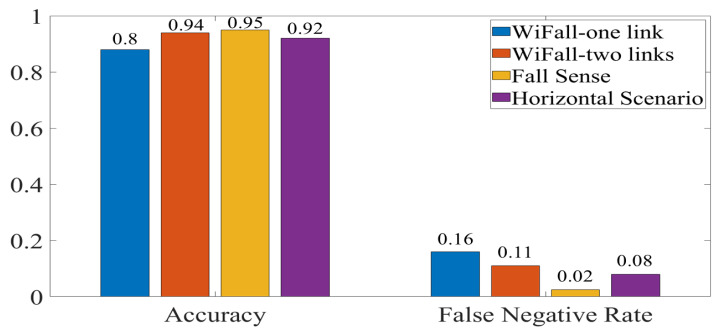
Comparison of Wifall, FallSense and HH scenario.

Figure 12 and Figure 13 show accuracy comparison between our experiments and two previously proposed systems. The comparison showed that classification accuracy was impacted by antenna orientation. Therefore, false negative rate was also affected. Although these systems used other features of the RF-WiFi signals for their recognition stage, the sensing stage was the common component in any system. WiFall-one link had a similar configuration to our system with only one $T_{X}$ and one receiver. Nevertheless, with the HH scenario, higher accuracy percentages were achieved. WiFall-two links have two receiver sensors, our accuracy percentage (Figure 13) was very close to their results. Finally, Fall sense reported a 3% higher accuracy than the accuracy achieved by our experiments at the cost of increasing the number of sensor. It should be noted that in our experiments only one sensor was used and with a careful consideration of antenna orientation, a increase of 12% was achieved. However, it was remarkable that orientation changes represented an important factor in the detection rate. Therefore, systems that considered these characteristics at the sensing stage could improve their accuracy and false-negative rate results. We expect that this configuration will help improve the difference between falls events and activities of daily living.

## 8. Discussion

Our preliminary results showed that the features extracted in the HH scenario corresponding to each participant have a trend in their values. The concordance of this indicates that under the same experimental conditions each person produced Doppler signatures different from those of the rest. Therefore, there is the possibility of characterizing the unique movement of each individual. This could close the gap with monitoring problems when there is the presence of more than one person moving near to the indoor environment. Furthermore, this would allow the proposed scenario to be expanded to less controlled environments such as monitoring in public places.

The results obtained in our experiments show that the HH scenario provides a better observation of Doppler shift that allows one to obtain a separable set of features through PCA. However, a set of different activities should be measured to ensure the correct classification of a fall. Human daily activities such as walking, walking waving arms or running, have a common characteristic, they produce a periodic Doppler shift as has been shown in [44]. However, seating and laying down are also non-periodic activities that do not produce a distinguishable Doppler shift as shown in a series of experiments measure with a 2.4 GHz radar [44]. In our future experiments, additional activities, such as walking towards or away from the stair, going up or down the stair and falling, should also be measured and analyzed to ensure that these activities can be distinguished from a fall. Additionally, feature extraction considering spatio-temporal evolution of the activities should be explored for classification of periodic and non-periodic activities. Early experiments including several activities has led us to expect that those activities can be distinguished.

Besides, aged population is rising due to advanced medicine and higher life quality. However, world population reports higher susceptibility to age-related diseases that can be addressed by health technology. Wireless Body Area Network (WBAN) has been explored as a healthcare technology that can monitor blood pressure, blood sugar level or other physiological variable that prevent catastrophic events [45]. WBAN architecture involves a dense network of sensor and actuators distributed in the body that interact with a coordinator that sends information outside the WBAN, a smartphone is normally used as a gateway. WBAN uses the 2.4 GHz ISM band to communicate between sensors and coordinator. There are several communication technologies used for intra-WBAN including Bluetooth, ZigBee, and Ultrawide band. The co-channel interference may occur due to the coexistence of WBAN with WiFi, ZigBee, Bluetooth, and cellular devices that share the same band. However, WiFi uses higher transmission power level than WBAN and consequently WiFi transmission dominates the medium [46]. Normally the gateway that sends information out of WBAN use WiFi, therefore, the interaction of WBAN with cloud-based services uses the same information channel that is used for the measure Doppler signatures. Quality of Service (QoS) measurements such as CSI or RSSI might be impacted by the interaction between these technologies, but Doppler signatures might not be affected.

Considering the above, it is possible to use the signals transmitted by commercial WiFi systems by analyzing Doppler signatures for fall detection without compromising coexistence. Furthermore, the analysis of the Doppler signatures allows the use of diversity techniques. In a WiFi system that transmits several pilot signals, the Doppler drifts will be replicated in each one. Therefore, the effects related to human movement can be compared in the same instant of time by more than one probe signal without the need to add a set of antennas to the system. This avenue has not been widely studied.

Finally, falling is a transient event that might be undetected due to the interruption of signal transmission. WiFi APs transmit their signals in bursts, interrupting the sensing process for short periods of time. Then, in the current transmission protocol that might cause a falling event to be undetected. Therefore, it should be considered to implement a continuous transmission mode with low power consumption that allows uninterrupted monitoring of the environment.

## 9. Conclusions

A study of the impact of antenna polarization in classification accuracy was performed. In order to determine this impact, an indoor WiFi platform was built with general-purpose equipment. The platform uses RF-WiFi signals to measure fluctuations caused by human movement without the need to carry any device. The design and development of the platform were presented as well as the fall experimentation protocol with volunteer subjects. At an operating frequency of 2.42 GHz we recorded the Doppler signatures on a series of spectrograms during testing. Two scenarios were proposed to characterize the effects caused by antenna orientation that impact the performance of the platform. The HH antenna scenario presented more prominent fluctuations during the tests. PCA was selected as a classification feature. It was shown that PCA component distribution shows difference in separability for the considered scenarios. A classification performed by SVM algorithm demonstrated that changing the orientation of the antennas can improve performance in detecting falling events. The impact of the antenna orientation in HH scenario shows a higher accuracy without increasing the number of sensors. Therefore, a careful consideration of antenna polarization should improve fall detection system design. When we compare these results with some of the systems that use a greater number of sensors, the relationship between accuracy and the false-negative rate had a similar performance. Therefore, our experiments showed that mitigation of the limitation of fall detection system based on a single sensor can be achieved by considering the effects of antenna orientation in the signal model. 

## Figures and Tables

**Table 1:** Approaches to fall detection systems.

Sensing Technique	Article and Year	Characteristics
Vision-Based	Zhang [4] (2020)	Needs to have a direct line of sight with the target, privacy concerns.
	Withanage [19] (2016)	
Wearable-Based	Hauth [5] (2021)	The device has to be worn by the user at all times.
	Zurbuchen [18] (2021)	
	Kerdjidj [20] (2020)	
Environment-Based	Liu [3] (2019)	Requires specialized and expensive implementation.
	Mun [2] (2015)	
Radio Frequency-Based	Y. Wang [13] (2017)	Provides data without the need to wear a device, it can be adapted to existing hardware.
	Huang [10] (2019)	
	W. Wang [11] (2017)	

**Table 2:** Transmission parameters in antennas.

Parameter	Value
Frequency	2.42 GHz
Return losses	32.4 dB
Impedance	50+j0.70
Voltage standing wave ratio	1.042

**Table 3:** Spectrum analyzer configuration.

Parameter	Value
Central frequency	2.42 GHz
Sweep time	271 ms
Sample points	1001
Frequency span	1 kHz

**Table 4:** Physical characteristics of test subjects.

Subject	Age	Gender	Height (m)	Weight (Kg)	Fall Tests
1	23	Female	1.61	60	20
2	24	Male	1.75	77	20
3	27	Male	1.72	73	20
4	38	Female	1.55	58	20
5	47	Female	1.55	65	20
6	48	Male	1.71	85	20
